# Evaluation of a Hypertension Surveillance System, Kenema Government Hospital, Sierra Leone, 2021

**DOI:** 10.5888/pcd20.220230

**Published:** 2023-03-23

**Authors:** Umaru Sesay, Gebrekrstos Negash Gebru

**Affiliations:** 1 Ministry of Health and Sanitation, Freetown, Sierra Leone; 2 Sierra Leone Field Epidemiology Training Program, Freetown, Sierra Leone

## Abstract

This observational study assessed key attributes of the hypertension surveillance system at Kenema Government Hospital, Kenema District, Sierra Leone. We administered semistructured questionnaires; reviewed hospital registers, patient charts, and the District Health Information Software database; and rated the implementation status of each attribute as poor (1–3), average (4–6), or good (7–10). Of the 7 attributes, simplicity, flexibility, and acceptability were good; stability was average, but timeliness, sensitivity, and data quality were poor. Overall, the usefulness of the hypertension surveillance system was poor, as it did not monitor hypertension trends, nor was it linked to public health action.

**Summary**

**What is known on this topic?**

In Sierra Leone, the hypertension surveillance system was established to monitor the trend and patterns of hypertension. However, its effectiveness is unknown, and the true burden of hypertension cannot be determined.

**What is added by this report?**

Although the system was relatively stable, this study highlights gaps in data collection, analysis, and dissemination. The system needs improvement to estimate the true burden of hypertension and to continue to monitor future trends.

**What are the implications for public health practice?**

Findings from this study can help to develop strategies and interventions to improve the surveillance system and guide hypertension control measures in Sierra Leone.

## Objective

Globally, in 2021, over 1 billion persons aged 30 to 79 years had hypertension (1), causing 7.5 million deaths (2). Sierra Leone, ranked 81 of 185 countries on the World Health Organization’s ranking of deaths caused by hypertension in 2022, has an age-adjusted death rate of 20.3 persons per 100,000 population (3). The government of Sierra Leone developed a national policy and strategic plan in 2013 for the prevention and control of noncommunicable diseases, including hypertension (4). However, hypertension prevalence among adults aged 40 years or older increased from 44% in 2014 to 50% in 2020 (5,6). Hypertension is reported in the District Health Information Software (DHIS2), a national database platform launched in 2012 for reporting, analysis, and dissemination of data for several health programs, including the hypertension surveillance system. All health facilities are required to report routinely collected hypertension data to DHIS2 (7). The main diagnostic and treatment facility for hypertension in the eastern region of Sierra Leone is the Kenema Government Hospital (KGH). The hospital reports monthly data to the monitoring and evaluation officer, who reports to the DHIS2 (Figure). However, anecdotal reports indicated the KGH hypertension surveillance system was not providing representative data to the DHIS2, and the true burden of hypertension was not clear (8). Identifying the true burden of hypertension in Sierra Leone could guide the prevention, control, and management of hypertension. This study assessed key attributes of the surveillance system to determine if it met its objectives of estimating and monitoring the burden of hypertension.

**Figure:**
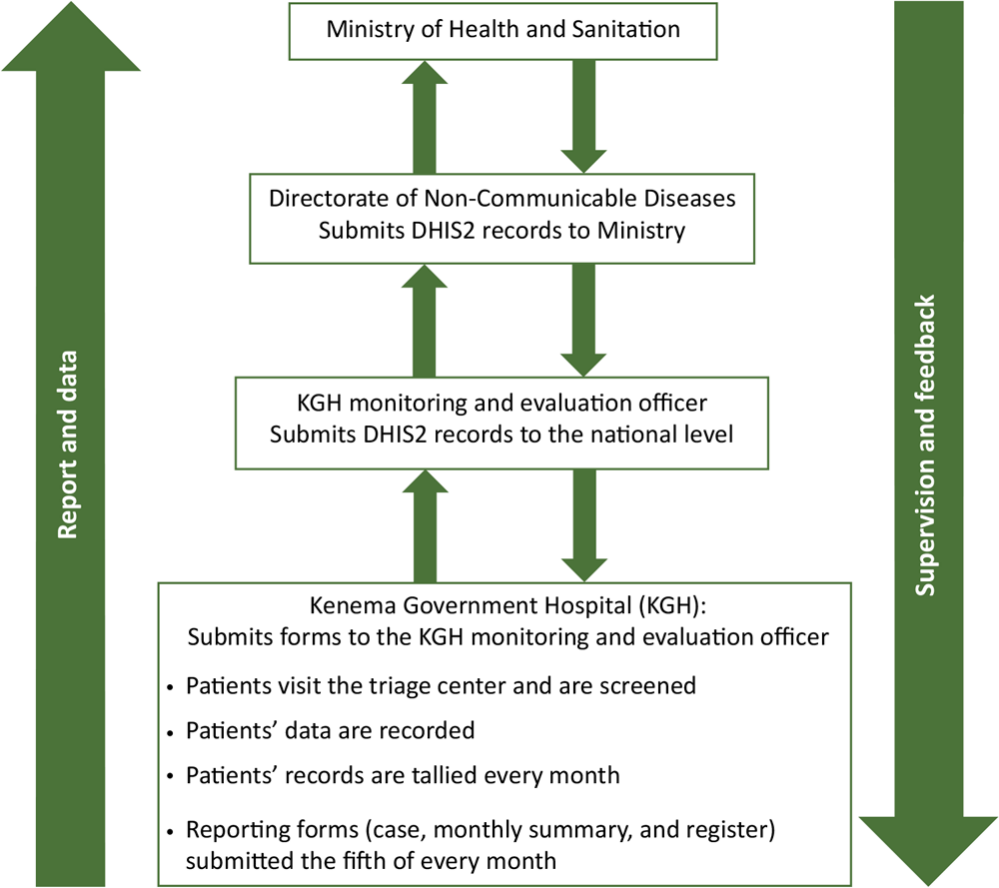
Process of the Hypertension Surveillance System in Kenema Government Hospital (KGH), Sierra Leone, 2021. Abbreviation: DHIS2, District Health Information Software.

## Methods

We conducted a descriptive cross-sectional study at the KGH from January through July 2021. KGH has 392 clinicians and 350 beds serving a population of 670,000 (9). All persons with symptoms of hypertension at other peripheral health units are referred to KGH for medical attention. We purposefully selected and interviewed 195 health care workers who met the inclusion criteria of at least 6 months of experience in managing hypertension patients at the hospital and consenting to participate in the interview. We conducted the interviews in a private setting, and participants were informed that their responses would be kept confidential. We also collected demographic variables of the health care workers.

We used the Updated Guidelines for Evaluating Public Health Surveillance Systems from the US Centers for Disease Control and Prevention (CDC) (10) to assess the hypertension surveillance system’s attributes. We used a semistructured questionnaire and observational techniques to collect qualitative data on usefulness, simplicity, flexibility, acceptability, and stability. We retrospectively reviewed and analyzed all available hospital registers, charts, and the DHIS2 database from January through December 2020 to collect quantitative data to assess data quality, sensitivity, and timeliness. We also explored independent data sources, including policies and regulations and gray literature from the Ministry of Health and Sanitation Sierra Leone websites to assess usefulness. We obtained permission from the hospital management to access the data from the hospital registers and charts. 

We used Epi Info version 7 software (CDC) to compute the frequencies and proportions of demographic and other quantitative variables. We used evaluation criteria to rate the quality and implementation status of each attribute on a scale from 1 to 10. Poor was 1 to 3, average was 4 to 6, and good was 7 to 10. To compute the evaluation scores, we calculated the proportion of total respondents who answered each question. We then averaged the evaluation scores for each attribute. We reported the evaluation scores for quantifiable data.

We obtained ethical clearance from the Sierra Leone Ethics and Scientific Review Committee (SLESRC), and permission to access data at KGH was granted by hospital management upon receiving the SLESRC clearance. Hospital management and all participating health workers signed a written consent form. To ensure confidentiality, we encrypted the Excel file where the responses were saved and used only codes during data analysis.

## Results

### Characteristics of participants

Of 195 health workers interviewed, 67.7% (132 of 195) were state-enrolled community health nurses, and 15.9% (31 of 195) were state-registered nurses. The median age of respondents was 32 years (interquartile range, 29–38 years), and 77.4% (151 of 195) were women. Of the health workers’ experience, 22.1% (43 of 195) had more than 10 years, 44.1% (86 of 195) had 5 to 9 years, and 33.8% (66 of 195) had between 1 and 4 years.

### Assessment of qualitative attributes

Simplicity was rated as good, with 68.7% (134 of 195) of respondents saying the case definition was simple to use in detecting hypertension cases. Regarding flexibility, the Ministry of Health and Sanitation introduced a new data tool for hypertension case reporting in September 2020. During our survey in 2021, 97.4% (190 of 195) of respondents stated that, although changes did occur in the case detection strategy, the changes did not affect the operation of the system. Therefore, flexibility was rated as good. Stability was rated as average, with 64.1% (125 of 195) of respondents saying they did not experience blood pressure monitors being out of stock. Acceptability was rated as good, as 100% (161 of 161) of records submitted by the deadline to the monitoring and evaluation officer were reported to the DHIS2. However, it was noted that the system relied on passive surveillance, which limited its capacity to detect and accurately monitor events over time and to identify any missing population groups in the entire district. A review of national, regional, and local rules, regulations, and policies, plus a gray literature review, identified no actions taken on data generated by the system (Table 1).

**Table 1:** Implementation Status of the Qualitative Attributes and Usefulness of the Hypertension Surveillance System in Kenema Government Hospital, Kenema District, Sierra Leone, January through July 2021

Qualitative indicators	Data source	Data collection method	Analysis	Implementation status
**Simplicity:** Ease with which health care workers identified hypertensive cases and if any steps or procedures were leading to delays in taking action or hindering the smooth functioning of the system.				**Good**
Case definition of hypertension	Health care workers	Key informant interview	68.7% (134 of 195) of respondents were able to explain the case definition in native language	
Hypertension reporting formats			72.8% (142 of 195) of respondents felt forms were easy to use	
Amount of time required to collect data from a person			71.8% (140 of 195) of respondents said they required 10 min or less to fill in a form for a hypertension case	
Awareness of the hypertension surveillance system flow			82.1% (160 of 195) of respondents were aware of the hypertension surveillance system flow	
**Flexibility:** If the system had adapted to any recent changes, such as changes in case definitions, reporting tools, data sources, diagnostic techniques, or reporting mechanisms.				**Good**
Changes in case detection	Health care workers	Key informant interview	68.7% (134 of 195) of the respondents said no changes occurred in the case detection strategy	
Changes in the reporting of hypertension records			97.4% (190 of 195) of the respondents said no changes occurred in the reporting of hypertension cases	
Addition of new data tools	Surveillance system record or announcement	Record review	A new data reporting tool was introduced in September 2020	
**Stability:** If there were any challenges to the consistent functioning of the system, for instance, inadequate reporting tools or personnel, the functionality of the DHIS2 software, internet connectivity, or availability of staff to carry out the surveillance system.				**Average**
Having no sphygmomanometers in stock	Health care workers	Key informant interview	64.1% (125 of 195) of respondents said sphygmomanometers were always in stock despite the COVID-19 disruption	
Breakdown in the operation of the system	Surveillance system record	Record review	The system did not experience any breakdown during the period under review	
**Acceptability:** Mean percentage of records reported on time per month to DHIS2, calculated as average of the total number of records recorded by the 5th of every new month divided by total number of cases per month multiplied by 100. Also, assessed the willingness of staff to work with the surveillance system and the completion rate of the report recorded in the register.				**Good**
Mean percentage of records reported to the DHIS2 by the monitoring and evaluation officer	DHIS2	Record review	100% (161 of 161)	
The availability and willingness of staff to carry out hypertension surveillance system	Observation	Observation	Staff shortage caused by priority of COVID-19 response activities	
Completeness of data variables assessed	Register	Observation	Most data elements had very low completion rates. Most of the variables included only age and sex	
Amount of time required to detect a case	Health care workers	Key informant interview	61.0% (119 of 195) of respondents said they required more than 1 visit to establish a diagnosis of hypertension in a participant	
**Usefulness:** Ability to meet the objectives of the system of monitoring hypertension trends and to use data for making public health decisions and taking actions.				**Poor**
Action taken on data generated	Health care workers	Key informant interview	68.2% (133 of 195) of respondents said no action was taken on the data	
Action taken by authorities	National/regional/local laws	Announcement between January 1 and December 31, 2020	No specific action was taken on the data generated to improve the performance of the hypertension surveillance system	
Action taken by authorities	Policies and regulations	Review of gray literature from the Ministry of Health and Sanitation, Sierra Leone website, between January 1 and December 31, 2020	No specific action was taken on the data generated to improve the performance of the hypertension surveillance system	
Collection of data on hypertension control	Health care workers	Key informant interview	56.4% (110 of 195) of respondents said they did not collect data on hypertension control	
Data analysis			63.1% (123 of 195) of respondents said they did not analyze hypertension data	

Abbreviation: DHIS2, District Health Information Software.

### Assessment of quantitative attributes

Timeliness was rated as poor; 78.8% (598 of 759) of records were not reported on time to the monitoring and evaluation officer. Sensitivity was rated as poor; the proportion of records submitted to the DHIS2 was 21.2% (161 of 759). Only follow-up cases were reported to DHIS2. Data quality was rated as poor; 4.5% (34 of 759) of records documented smoking history, 2.8% (21 of 759) reported weight, and no record documented physical activity and lipid profiles (Table 2).

**Table 2:** Implementation Status of the Quantitative Attributes of the Hypertension Surveillance System in Kenema Government Hospital, Kenema District, Sierra Leone, January through December 2020

Quantitative indicators	Data source	Data collection method	Analysis	Implementation status
**Timeliness:** The time interval between the various steps in the surveillance system or any delays in taking action.				**Poor**
Proportion of records not reported on time to the monitoring and evaluation officer by those in charge at the hospital	Register	Record review	78.8% (598 of 759) of records were not reported on time to the monitoring and evaluation officer	
Median time to fill in a hypertension patient form	Health care workers	Key informant interview	Average time of 10 min (range, 6–17 min) to fill in the form	
Proportion of health workers who did not receive timely feedback on hypertension surveillance system activities			57.4% (112 of 195) of the respondents said they did not receive timely feedback from supervisor on the performance of the system	
**Sensitivity:** Determining the proportion of cases detected by the DHIS2 system per health facility register.				
Proportion of cases detected by system vs records in register	Register and DHIS2	Record review	21.2% (161 of 759)	**Poor**
**Data quality:** Determining the proportion of filled variables in the register.				
Proportion of records that include age	Register	Record review	100% (759 of 759)	**Poor**
Proportion of records that include sex			100% (759 of 759)	
Proportion of records that include smoking status			4.5% (34 of 759)	
Proportion of records that include weight			2.8% (21 of 759)	
Proportion of records that include physical activity			0% (0 of 759)	
Proportion of records that include lipid profile			0% (0 of 759)	

Abbreviation: DHIS2, District Health Information Software.

## Discussion

Of the 7 attributes we assessed, data quality, sensitivity, and timeliness were poor; stability was average; and simplicity, flexibility, and acceptability were good. The overall usefulness of the KGH surveillance system was rated as poor, as it did not fully meet its objective to monitor trends and epidemiologic patterns of hypertension and was not linked to public health action.

The evaluation revealed that the sensitivity of the surveillance system was lower than that of the Ebola surveillance system (21.2% vs 88.5%) in a study conducted at the Tonkolili district, Sierra Leone (11). Because only follow-up cases were reported to the DHIS2, the true burden of hypertension, if estimated by using data generated from the DHIS2, might be a lower estimate.

Even though the system was noted as simple for staff to use, the data quality was poor. Most of the known risk factors, such as smoking history, weight, physical activity, and lipid profiles, were not captured in hospital records. These missing data could possibly impair decision making or program planning for specific interventions. The lack of data could be due to a shortage of effective oversight by supervisors. The supervisors did not monitor the data process within the hospital, nor did they provide regular feedback to health staff on the system’s performance. Although the system continuously functioned during the study period, the stability of the system was affected by the COVID-19 pandemic. Both human and financial resources were diverted to respond to COVID-19 prevention and control activities. This study also revealed that, even though KGH is the main referral hospital in 3 districts for persons presenting with symptoms of hypertension, the hypertension surveillance system’s reliance on passive surveillance limits its capacity to detect the occurrence of cases at the community level. This implies that the system is not generating data that can reflect the true burden and pattern of hypertension in the population under surveillance.

The main limitations of the study included a purposefully selected sample for assessing the qualitative attributes. Also, because of the lack of available data we were not able to quantitatively assess the predictive value positive and representativeness of the surveillance system. Although everyone seeking care at KGH was triaged to improve patient health outcomes, the number of persons screened, including those referred by health workers or self-referred, was unavailable.

When compared with communicable disease surveillance systems, only minimal support is provided to improve the hypertension surveillance system in many low-income countries, including Sierra Leone. Lack of funding for staff recruitment, training, and logistics presents a substantial challenge to the hypertension surveillance system’s ability to generate data that are supported by evidence and used in public health.

In summary, the overall score for the surveillance system was poor. The disease burden could not be determined by using the generated data, and the trend and pattern of hypertension in KGH could not be monitored. The system’s low sensitivity might have limited its capacity to detect, estimate, and monitor the burden of hypertension in the population. Its usefulness was limited as it fell short of its objectives and the data generated were not used for decision making or action. The general process of data collection and recordings should be expanded, and a supervision system should be put in place to strengthen the performance of the system. Therefore, we recommend that data collection, input of data into the system, and analysis of data be strengthened through increased hiring and training. Supervision should be increased to focus on entering and reporting data to the DHIS2.
